# Presurgical Pulmonary Function Tests in the First Few Days of Life in Neonates with Congenital Heart Disease, A Pilot Study

**DOI:** 10.21203/rs.3.rs-3938413/v1

**Published:** 2024-02-13

**Authors:** Cindy McEvoy, Hayden Leeds, Ashok Muralidaran, Alicia Johnson, Diane Schilling, Kseniya Parkhotyuk, Irving Shen

**Affiliations:** Oregon Health & Science University

**Keywords:** Newborn pulmonary function, congenital heart disease, respiratory resistance, respiratory compliance

## Abstract

**Objective:**

To compare early pulmonary function tests (PFTs) in neonates with critical congenital heart disease (CHD) compared to a historical reference group.

**Design:**

Infants > 37 weeks gestation with critical CHD were studied within the first few days of life and prior to cardiac surgery and compared to data from a published reference group. Passive respiratory resistance (Rrs) and compliance (Crs) were measured with the single breath occlusion technique following specific acceptance criteria. The study was powered for a 30% difference in Rrs.

**Results:**

PFTs in 24 infants with CHD were compared to 31 historical reference infants. There was no difference in the Rrs between the groups. The infants with CHD had a significantly decreased Crs (1.02 ± 0.26 mL/cmH2O/kg versus 1.32 ± 0.36; (p < 0.05; mean ± SD)).

**Conclusions:**

Further prospective studies are required to quantify early PFTs in infants with CHD of different phenotypes.

## Introduction

There are approximately 40,000 children born each year in the United States with congenital heart disease (CHD), and about 25% are born with a critical heart defect that will require surgery or other medical procedures in their first year of life.^1^ These defects can be further subdivided into those leading to increased or decreased blood flow to the lung, i.e. pulmonary blood flow. Since the lung is a network of air spaces and blood vessels, the volume of blood flowing or not flowing through the lungs has an intrinsic impact on the mechanical properties of ventilation which can have large implications on the infant’s clinical course and overall management. The cardiopulmonary system must be often be surgically altered in the attempt to either increase or decrease pulmonary blood flow depending on the physiology of the heart lesion.^2,3^

Prior studies have described abnormalities in pulmonary function tests (PFTs) in children with CHD, including, restrictive, obstructive, and diffusion abnormalities.^4^ Cardiac lesions with left to right shunting and associated increased pulmonary blood flow (Qp), often develop pulmonary edema with PFTs demonstrating increased total respiratory resistance (Rrs) and decreased dynamic compliance.^5–8^ Conversely, cardiac lesions with associated low Qp have demonstrated a postoperative increase in respiratory resistance compared to preoperative measurements. These change were associated with the improvement in Qp.^9^ Altered preoperative PFTs have been shown to be associated with a postoperative prolonged length of intubation and an increased stay in the intensive care unit. Thus, the majority of prior work has focused on the use of these measures of pulmonary function to predict post-operative outcomes.^10,11^

Although the above studies have demonstrated alterations in PFTs prior to and following cardiac surgery in older infants and children, to the best of our knowledge, no study has quantified PFTs in neonates with CHD with single and two ventricle physiology within the first few days of life as a reflection of the possible in utero effects of CHD on lung development. Thus, the objective of this study was to compare PFTs, including Rrs and passive respiratory compliance (Crs), performed within the first few days of life in non-sedated neonates with critical CHD compared to a reference group of healthy term neonates. We hypothesized that neonates with congenital heart disease would have altered PFTs compared to healthy, term neonates. Additionally, we hypothesized that differences would exist within the CHD cohort related to the presence of single versus double ventricle physiology.

## Materials/Subjects and Methods

The Oregon Health & Science (OHSU) Institutional Review Board approved this prospective cohort study, which included a study cohort of neonates with CHD and a reference cohort of healthy term neonates.^12^ The study was performed in the Neonatal Intensive Care Unit (NICU) and normal newborn nursery at OHSU. Demographic and clinical data were collected prospectively for both cohorts. Informed consent was obtained for all enrolled participants. Eligibility criteria for the study cohort included neonates born at greater than 37 weeks of gestational age with a critical CHD that would likely require surgery in the first year of life. These eligible lesions included: hypoplastic left heart syndrome, hypoplastic right heart syndrome, transposition of the great arteries, pulmonary atresia, coarctation of the aorta, truncus arteriosus, tetralogy of fallot, double outlet right ventricle, atrioventricular canal, aortopulmonary window, and Shone’s complex. The CHD study cohort was further subdivided into those neonates with heart lesions predisposing them to single ventricle physiology and those with two ventricle physiology. We aimed to enroll an equal number of subjects within each group in order to perform a secondary analysis of PFTs based on the type of cardiac lesion present, single versus two ventricle physiology. Exclusion criteria were neonates: born at less than 37 weeks gestation, with known or diagnosed congenital pulmonary abnormalities, such as congenital diaphragmatic hernia, prolonged rupture of membranes, or culture proven sepsis.

The reference cohort of healthy term neonates consisted of appropriate for gestational age neonates delivered at > 37 weeks of gestation who had required no supplementation oxygen or continuous positive airway pressure (CPAP) and were studied within 72 hours of life and prior to discharge. Exclusion criteria for the reference group included: maternal history of smoking; documented sepsis; multiple congenital anomalies; history of oligohydramnios; congenital heart disease; evidence of respiratory distress syndrome.^12^ These infants were previously studied at our institution by the same pulmonary function technician/respiratory therapist (DS) who also performed the current PFTs in the study group with CHD.

The PFTs were performed with the neonatal/infant pulmonary function cart (SensorMedics 2600; SensorMedics Inc, Yorba Linda, California) that had been used to study the healthy reference group. The measurements were done within the first few days of life with the infants breathing through a face mask that was connected to a 3-way valve, or if intubated, through the endotracheal tube. Flow volume loops were collected with inspiratory and expiratory volumes within 15% and tidal volumes calculated. Rrs and Crs were measured with the single-breath occlusion technique.^13,14^ During these measurements, the airway was briefly occluded at end inspiration until an airway pressure plateau was observed and the Hering–Breuer reflex was invoked.^14^ The linear portion of the passive flow-volume curve was identified, and a regression line was drawn for the best fit. From the intercepts on the flow and volume axes, Crs and respiratory resistance (Rrs) were calculated. Acceptance criteria as per the ATS/ERS were applied and at least 10 breaths were accepted with a coefficient of variation of < 20%.^14^

The primary outcome was the difference in Rrs measurements between the infants with CHD and the healthy reference group. We hypothesized based on previous data from the historical controls,^12^ about 24 infants with congenital heart disease (CHD) were needed to show a 30% difference in Rrs with an alpha of 0.05 and power of 80%. We also compared Crs measurements between the two groups of infants as a secondary outcome. The Rrs and Crs in infants with single versus two ventricle physiology were also compared. PFT outcomes were compared between the CHD and reference group with the Wilcoxon rank sum test or the two-sample t-tests, as appropriate. Differences among the two groups of congenital heart defects were reported using descriptive statistics.

## Results

**Figure 1:**
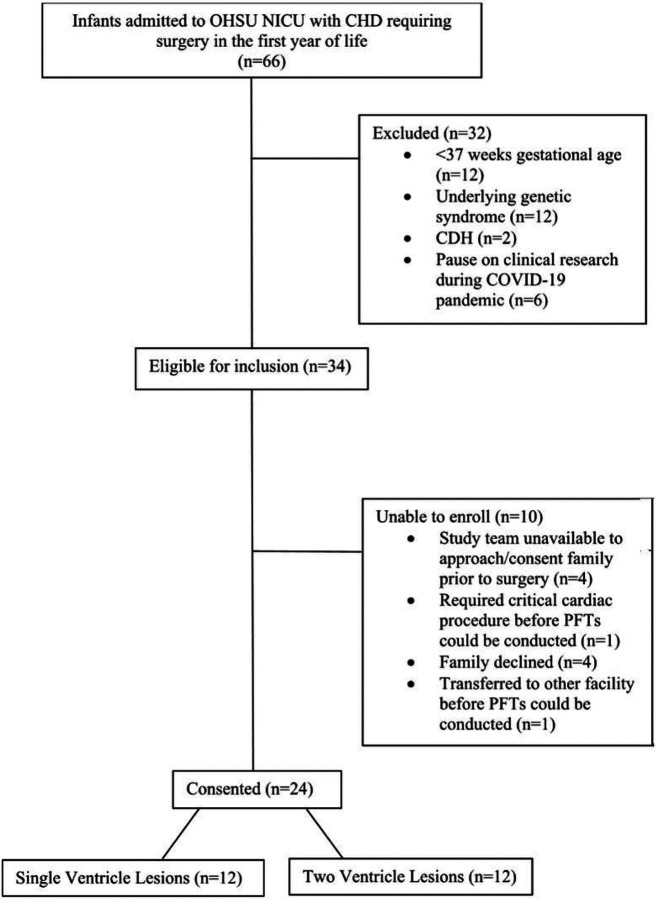
Legend is not included with this version

From April, 2019 through February, 2022, 66 infants with critical CHD were admitted to the OHSU NICU, 34 met eligibility criteria, and 24 were consented into the study (Fig. 1). The most common diagnosis in the study population was hypoplastic left heart syndrome (25%). Twelve infants had single ventricle physiology and 12 had CHD with two ventricle physiology (Table 1). The 24 neonates with CHD (67% male) had a mean gestational age (GA) of 38.6 weeks and a mean birth weight (BW) of 3281 grams (Table 2). The 31 reference neonates (42% male) had a mean GA of 39.3 weeks and BW of 3561 grams (Table 2). The CHD cohort had clinical echocardiograms, done within the first 72 hours of life, that showed 33.3% with exclusive left to right shunting, 25% with exclusive right to left shunting, and 41.7% with mixed shunting.

The CHD cohort was studied at a median of 48 hours of life and the healthy, term reference cohort at 24 hours of life (Table 3). All participants were studied during non-sedated, quiet sleep. Two neonates with CHD were on mechanical ventilation at the time of PFT, one for apnea and one for hypoventilation. All other participants in both groups were on room air and the average respiratory rate of the CHD cohort being 55 breaths per minute. There was no difference in Rrs or tidal volume between the CHD cohort compared to the healthy reference group, however, the CHD cohort had a significantly decreased Crs at 1.02 ± 0.26 mL/cmH2O/kg versus 1.32 ± 0.36 in the reference group (p < 0.05; mean ± SD) (Table 3).

The PFTs in the CHD cohort were further evaluated in terms of single versus two ventricle physiology. The Rrs in the 12 single ventricle patients was 0.047 cmH2O/mL/sec (0.032, 0.055) [median, IQR], compared with 0.055 cmH2O/mL/sec (0.039, 0.071) in the 12 two ventricle patients. The Crs was 1.08 mL/cmH2O/kg (0.27) [mean (SD)] in the single ventricle versus 0.97 mL/cmH2O/kg (0.26) in the two ventricle physiology group. The expiratory volume was 5.73 mL/kg (1.32) [mean (SD)] in the single ventricle versus 6.15 mL/kg (1.51) in the double ventricle physiology group

## Discussion

In this pilot study, there was no difference in the Rrs measured preoperatively in the first few days of life in 24 neonates with critical CHD (including both single and two ventricle physiology) compared to 31 healthy term reference neonates. However, the neonates with CHD had a decreased Crs compared to the healthy term reference neonates. This confirms findings from previous studies in older infants and children that have demonstrated decreased compliance, particularly those with lesions with increased pulmonary blood flow.^5–8^ The decrease in Crs could reflect alterations in pulmonary blood flow, but 22 of the 24 neonates in the CHD cohort were stable on room air at the time of testing and only one third had exclusive left to right shunting by echocardiogram. Additionally, the early postnatal age at which they were tested would likely be prior to a decrease in the pulmonary vascular resistance, which usually occurs within the first few weeks of life.^2^ The decreased Crs could also reflect in utero developmental changes that may occur in infants with CHD.^15^ To our knowledge, this study reports the earliest measurements of pulmonary function in non-sedated, non-intubated neonates compared to a cohort of healthy term reference neonates and increases our knowledge of early altered PFTs in neonates with CHD.

Other investigators have reported increased Rrs and decreased Crs in older infants and children with CHD, particularly those with increased pulmonary blood flow.^5,8,9^ Stayer et al performed serial measurements of Rrs and dynamic compliance in 106 infants undergoing cardiac surgery with cardiopulmonary bypass at a mean of 4.3 months of age.^5^ The majority of the surgical procedures were bidirectional cavopulmonary shunt, stage 1 correction of hypoplastic left heart syndrome, atrioventricular canal repair and tetralogy of fallot repair. This study demonstrated a decrease in Rrs after the correction of the cardiac lesions with subsequent increased pulmonary blood flow.^5^ The authors posited that possible explanations for the preoperative increased Rrs and decreased Crs included enlarged pulmonary vasculature leading to compromised gas exchange, pulmonary hypertension and pulmonary edema.^5^ Agha et al studied 30 infants with left to right shunt congenital acyanotic heart diseases at a mean of 10.5 months and demonstrated a significant increase in Crs and decrease in Rrs six months after surgery, as compared to preoperative measurements. In this study echocardiographic parameters of pulmonary vascular engorgement and pulmonary artery pressure were closely associated with infant PFT measures.^8^

Although we did not identify a difference in Rrs in the infants with CHD compared to the historical reference group, this may reflect the early timepoint of our measurement. Other studies using similar PFT techniques in older infants and children have reported differences in Rrs, particularly before and after surgery.^5,8,9^ Additionally, the measures applied in our study primarily quantify the Rrs of the larger central airways, rather than the peripheral airways. The application of other measures of pulmonary function, such as neonatal forced oscillometry,^16–18^ may be a more pragmatic and sensitive measure of peripheral airway resistance. Additionally, early measurements of lung diffusion capacity in infants may be used,^19^ however, this is not currently feasible in the NICU.

Our study has a number of strengths including the measurement of neonatal PFTs in the first few days of life in infants with CHD prior to surgical intervention and the unique ability to perform standardized neonatal PFTs by an experienced research team following American Thoracic Society and European Respiratory Society criteria for acceptability.^14^ In addition, we have used neonatal PFTs to quantify the effects of a variety of medications and interventions, such as different permutations of antenatal steroids on preterm infant PFTs and vitamin C supplementation in pregnant smokers to mitigate the effects of nicotine on lung development. We have demonstrated that these measurements correlate with clinical outcomes.^13,20^

There were several limitations to our study. This is a pilot study performed at a single center with a small sample size, however, this study was powered to demonstrate a 30% difference in Rrs. ideally the healthy reference cohort would have been studied concurrently to the CHD cohort, but this was not possible due to budget limitations coupled with intermittent study shutdowns due to the COVID-19 pandemic. This limitation is minimized by the use of the same standardized PFT protocol, same PFT testing equipment, and same PFT technician (DS) performing the testing for both cohorts. We were unable to obtain FRCs, an important measure of pulmonary function, for many of the single ventricle patients because of concerns that the brief administration of 100% oxygen could exacerbate any potential pulmonary overcirculation.

There have been a limited number of studies examining potential altered lung development in infants with CHD. A recent study of lung samples from humans and animals with reduced Qp physiology demonstrated impaired alveolarization and vascularization compatible with a pulmonary dysplasia.^15^ A prior study by Simonato et al found a difference in the composition of epithelial fluid in patients with CHD at the time of surgery, suggesting there may be differences in the biological profile of such patients that may account for differences in pulmonary mechanics.^21^ These studies, along with the findings in our study, suggest that there may be multiple factors, including pulmonary blood flow, associated with the changes in pulmonary mechanics in neonates with critical CHD, necessitating future investigations.

Our CHD cohort included an equal number of patients with single and two ventricle physiology. Due to the small numbers in each cohort, we were unable to draw any statistically significant conclusions about differences that exist amongst the groups. Our future goal is to quantify PFTs in a larger cohort of single versus two ventricle patients, as we hypothesize that baseline PFTs may have implications on the eventual management of these lesions. Specifically, infants with single ventricle physiology undergo a series of staged procedures culminating in the Fontan operation in which all venous return flows passively through the lungs, before returning to the heart.^3^ By understanding baseline differences in pulmonary mechanics within this population, our goal is to guide future research exploring whether these changes contribute to efficiency of this passive circulation and clinical outcomes.

In conclusion, this novel pilot study demonstrates an early difference in neonatal PFTs, primarily, reduced Crs in neonates with congenital heart disease. There was no statistically significant difference in Rrs at this early measurement timepoint. Further studies are needed to quantify early PFTs in infants with CHD with single and two ventricle physiology with the goal of more informed planning of subsequent corrective surgeries.

## Figures and Tables

**Table 1:** Characteristics of Neonates with Congenital Heart Disease

Primary Cardiac Diagnosis (N = 24)	N (%)
Hypoplastic Left Heart Syndrome (Single Ventricle)	6 (25)
Hypoplastic Right Heart (Single Ventricle)	4 (16.7)
Transposition of the Great Arteries (Two Ventricle)	4 (16.7)
Pulmonary Atresia (Single Ventricle)	2 (8.3)
Coarctation of the Aorta (Two Ventricle)	2 (8.3)
Truncus Arteriosus (Two Ventricle)	1 (4.2)
Tetralogy of Fallot (Two Ventricle)	1 (4.2)
Double Outlet Right Ventricle (Two Ventricle)	1 (4.2)
AV Canal (Two Ventricle)	1 (4.2)
AP Window (Two Ventricle)	1 (4.2)
Shone’s Complex (Two Ventricle)	1 (4.2)

**Table 2:** Demographics of Neonates with CHD and Reference Neonates

Variable	Neonates with CHD (N = 24)	Reference Neonates (N = 31)
Gestational age (weeks) – Mean (SD)	38.6 (1.06)	39.3 (0.94)
Birth weight (g) – Mean (SD)	3281 (425.2)	3561 (435.8)
Birth length (cm) – Mean (SD)	49.8 (1.95)	51.2 (2.47)
Sex – N (%)		
M	16 (66.7)	13 (41.9)
F	8 (33.3)	18 (58.1)
Race/Ethnicity – N (%)		
Caucasian	16 (66.7)	17 (54.8)
Hispanic	3 (12.5)	13 (41.9)
Black	2 (8.3)	1 (3.2)
Asian	2 (8.3)	0 (0)
Pacific Islander	1 (4.2)	0 (0)

**Table 3:** Age at PFT and Rrs values showing median (Q1, Q3), all other values showing mean (SD); Comparisons of PFTs by Wilcoxon rank sum test or by two sample t-test as appropriate

Variable	Neonates with CHD (N = 24)	Reference Neonates (N = 31)
Age at PFTs (hours of life)	48 (48,72)	24 (24,72)
On room air at time of PFT	22/24	24/24
Rrs (cm H2O/mL/sec)	0.05 (0.035, 0.057)	0.04 (0.036, 0.048)
Crs (mL/cmH2O/kg)	1.02 (0.26)	1.32 (0.36)*
Total Crs (mL/cmH2O)	3.36 (0.88)	4.66 (1.17)^†^
Tidal volume (mL/kg)	5.94 (1.40)	6.00 (1.11)

* p < 0.05

† Comparative analysis not performed, as already performed for Crs and found to be statistically significant

## Abbreviations

CHD: Congenital heart disease
Crs: Passive respiratory system compliance
FiO_2_: Fractional inspired oxygen concentration
FRC: Functional residual capacity
PFTs: Pulmonary function tests
Qp: Pulmonary blood flow
Rrs: Passive respiratory system resistance
